# Low-grade infections as a potential contributor of arthrofibrosis following total knee arthroplasty

**DOI:** 10.5194/jbji-10-303-2025

**Published:** 2025-08-13

**Authors:** Lucía Henríquez, Ander Uribarri, Ignacio Sancho, Maria Eugenia Portillo

**Affiliations:** 1 Clinical Microbiology Service of the University Hospital of Navarra (HUN), Irunlarrea 3, 31007, Pamplona, Navarra, Spain; 2 Traumatology Service of the University Hospital of Navarra (HUN), Irunlarrea 3, 31007, Pamplona, Navarra, Spain; 3 Sanitary Research Institute of Navarra (IdiSNA), Irunlarrea 3 31007, Pamplona, Navarra, Spain

## Abstract

Arthrofibrosis is a well-known complication of arthroplasty. However, the underlying causes that may lead to this pathology are unclear. A possible relationship between undiagnosed low-grade infections and the development of arthrofibrosis has been proposed (Ibrahim et al., 2020). This study aims to investigate whether colonization of the joint by low-virulence pathogens may cause arthrofibrosis.

## 1 Introduction

Arthrofibrosis is a rare but highly limiting complication characterized by the excessive production of fibrous scar tissue, which can occur following trauma or surgery on the knee joint (Ramos et al., 2023). Clinically, it presents as a limitation in extension and/or flexion, often accompanied by pain, functional impairment, and claudication. Stiffness is multifactorial, and numerous risk factors (pre-operative, intraoperative, and post-operative) have been identified. However, the exact aetiology remains poorly understood. Previous studies have suggested potential contributing factors, including genetic predisposition, inadequate rehabilitation, and underlying infections (Ibrahim et al., 2020).

In recent years, research has attempted to establish a link between arthrofibrosis and low-grade peri-prosthetic joint infections (PJIs); however, a definitive causal relationship between these two conditions has not yet been demonstrated (Brückner et al., 2019). This study aims to investigate whether colonization of the joint by low-virulence pathogens may be related to the development of arthrofibrosis in patients undergoing total knee arthroplasty (TKA).

## 2 Methods

Nineteen patients who underwent TKA at our centre, University Hospital of Navarre (HUN) ( ≈ 1000 beds), between 2019 and 2023 were studied. Arthrofibrosis was diagnosed according to Kalson's criteria and all of the patients required additional surgeries to restore mobility. A complete analysis was performed to discard infection. C-reactive protein (CRP) and pre-operative and post-operative range of motion (ROM) were recorded. In those cases in which open surgery was performed, four tissue biopsy samples were intraoperatively collected in native vials in 14 of the 19 cases. Tissue specimens were sent to the anatomical pathology (AP) and microbiology departments. For microbiological culture, tissues were inoculated in Schaedler agar enriched with 5 % sheep blood (BioMérieux, Marcy L'Etoile, France), PolyViteX agar (BioMérieux, Marcy L'Etoile, France), and thioglycollate broth (BBL-enriched thioglycollate medium with vitamin K and hemin; Beckton Dickinson and Company, USA). Aerobic cultures were incubated at 37 °C for 1 week, and anaerobic cultures were incubated for 2 weeks. PJI criteria followed the European Bone and Joint Infection Society (EBJIS) 2021 guidelines (Table 1). Qualitative variables were assessed using chi-square ( $\chi^{2}$ ) tests. Statistical significance was considered for p values < 0.05.

**Table 1:** Assessment of peri-prosthetic joint infection (PJI) according to EBJIS 2021. The main criteria did not allow confirmation of the suspicion.

EBJIS criterion	Findings	Interpretation
Sinus tract communicating with the joint^*^	None observed	PJI not confirmed
Purulence around the prosthesis^*^	None observed	PJI not confirmed
Microbiological evidence ( ≥2 cultures with the same organism)^*^	No growth/not concordant	PJI not confirmed
Elevated CRP	Within the normal range	PJI not confirmed
Elevated synovial WBC or PMN percentage	Not elevated	PJI not confirmed
Positive histology (neutrophils ≥5 /HPF in ≥5 fields)	No significant neutrophilic infiltrate	PJI not confirmed
Positive alpha-defensin or leukocyte esterase test	Not performed/negative	PJI not confirmed

Note: ^*^ major criteria. All patients included in the study were classified as “not infected” based on the absence of major criteria and the lack of sufficient minor criteria to suggest infection according to the EBJIS 2021 definition.

## 3 Results

Of the 14 patients from whom intraoperative samples were obtained, 7 (50 %) were male. The mean age was 71, with only two patients under 65 (62 and 63 years old). The predominant co-morbidities in our cohort were dyslipidaemia (71.4 %) and arterial hypertension (64.3 %), followed by diabetes mellitus 2, obesity, and cardiovascular disease (21.4 %). Additionally, three patients had a history of hiatus hernia, and one patient had osteoporosis.

In none of the patients was PJI suspected or diagnosed. The pre-operative CRP was >10 mg L^−1^ in nine (64 %) of the patients. Among them, four out of six (67 %) had positive cultures. In the remaining two patients with microbiological isolation, CRP was not determined pre-operatively. Samples for AP were sent in only six cases; in none of them were the findings suggestive of infection. Eight patients (57 %) required subsequent surgery, 67 % of them with positive cultures and 50 % of them with no microbiological isolations ( p>0.05 ). The average pre-operative ROM was 10–70°, while the average post-operative ROM was 5–100°. At 1 year, 64.3 % of the patients had a greater ROM compared to pre-surgery. One of the patients passed away.

Low-virulence microorganisms were identified in 6 out of 14 cases (42.9 %) (Table 1). Microbiological cultures yielded four coagulase-negative staphylococci, one *Streptococcus* viridans, and one *Cutibacterium acnes*, each in a single positive culture. No statistically significant differences were observed in the follow-up between culture-positive and culture-negative patients in terms of ROM ( p>0.05 ). The clinical and laboratory parameters for all patients included in the study are summarized in Table 2.

**Table 2:** Demographic and microbiological data of the 14 patients with arthrofibrosis from whom microbiological samples were taken.

	Number of patients	
	( n=14 )	
Demographics		
Mean age (SD)	71 (4.5)	
Male sex	7 (50 %)	
Co-morbidities		
Dyslipidemia	10 (71.4 %)	
Arterial hypertension	9 (64.3 %)	
Type-2 diabetes mellitus	3 (21.4 %)	
Biomarkers		
PCR > 10 mg L^−1^	9 (64 %)	
Microbiology		
Negative culture	8 (57.1 %)	
Positive culture	6 (42.9 %)	
	4	Coagulase-negative staphylococci Three *Staphylococcus epidermidis* One *Staphylococcus warneri*
	1	*Streptococcus* viridans group *Streptococcus oralis*
	1	*Cutibacterium acnes*
Outcome		
Average pre-operative ROM	10–70	
Average post-operative ROM	5–11	
Subsequent surgery	8/14 (57 %)	
Culture-positive patients	4/6 (67 %)	
Culture-negative patients	4/8 (50 %)	
Exitus	1 (7.1 %)	

ROM: range of movement.

**Table 3:** Microbiological, biochemical, anatomical pathology, and clinical data of patients grouped according to their microbiological outcomes.

Patient	Microbiology	Pre-PCR	AP	Subsequent	PRE-ROM	POST-ROM	PRE-ROM	POST-ROM
	culture	(mg L^−1^)		surgery			(average)	(average)
1	*S. warneri*	44	YES	NO	20–85	0-95	10–65	0–95
2	*S. epidermidis*	22	NO	YES	0–40	5–90		
3	*S. epidermidis*	200	NO	YES	0-80	0–80		
4	*S. epidermidis*	–	YES	NO	15–75	0–130		
5	*S. oralis*	63	NO	YES	0–30	0–95		
6	*C. acnés*	–	YES	YES	30–80	5–80		
7	NEG	19	NO	YES	0–70	5–120	7–70	0–100
8	NEG	112	NO	YES	0–80	0–90		
9	NEG	84	YES	YES	–	0–80		
10	NEG	26	NO	NO	35–95	–		
11	NEG	–	YES	NO	0–45	0–90		
12	NEG	39	YES	NO	5–85	0–110		
13	NEG	–	NO	YES	10–60	0–90		
14	NEG	–	NO	NO	0–70	0–100		
15	NS	–	NO	NO	20–100	5–100		
16	NS	–	NO	NO	20–70	5–85		
17	NS	3	NO	NO	30–85	0–120		
18	NS	–	NO	NO	5–80	0–90		
19	NS							

NS: no sample. NEG: negative. Pre-PCR: pre-operative PCR. AP: pathological anatomy. PRE-ROM: pre-operative range of movement. POST-ROM: post-operative range of motion.

## 4 Discussion

TKA is generally a successful and cost-effective procedure; however, adverse outcomes like arthrofibrosis can lead to costly follow-up procedures (Pfefferle et al., 2014). The clinical aetiology of acquired idiopathic stiffness – and, by extension, arthrofibrosis – is multifactorial (Cheuy et al., 2017). Understanding its potential risk factors and underlying causes is essential for the effective management of this complication. In our patient cohort, we found that dyslipidaemia and arterial hypertension were the main co-morbidities.

The pathophysiology of arthrofibrosis involves the activation and proliferation of myofibroblasts, which deposit type-I collagen in response to a proinflammatory environment (Ramos et al., 2023). Although the exact mechanisms by which these factors lead to arthrofibrosis are not totally clear, it is known that cell growth, differentiation, and death controlled by various cytokines and growth factor signals is disrupted, directly affecting tissue homeostasis (Cheuy et al., 2017). A recent study based on bioinformatics analysis identified transforming growth factor beta receptor 1 (TGFBR1) as a potential biomarker for arthrofibrosis (Chen et al., 2021). Other authors propose component malrotation as a potential contributor to stiffness, suggesting that it may significantly impact post-operative ROM (Amanatullah et al., 2019). In this context, we hypothesize that this sustained inflammatory environment may be induced in response to a low-grade infection prolonged over time.

Although none of the patients met the criteria for the diagnosis of confirmed PJI, our findings suggest a potential link between arthrofibrosis and low-virulence microorganism infections. However, this possible infectious aetiology did not correlate with clinical outcomes during follow-up. In nearly half of the patients with biopsy cultures (42.9 %), a low-virulence microorganism was isolated in only one tissue culture. As all findings were negative according to the EBJIS 2021 criteria, the likelihood of true infection was low in all cases. However, the fact that more than half of the patients had CRP levels greater than 10 mg L^−1^ may support the hypothesis of a subclinical or low-grade infectious process. Of the seven isolates, three were identified as *S. epidermidis *and one as *C. acnes*, microorganisms commonly associated with unexpected positive cultures during revision total hip arthroplasty and TKA (Kayani et al., 2024).

Given the small sample size and the exclusive use of conventional culture methods, further studies with larger cohorts, incorporating a control group and molecular biology techniques, are needed to clarify the potential role of low-virulence microorganisms in arthrofibrosis. Conventional microbiological techniques, such as a single positive tissue culture with common skin contaminants, may yield false-negative results in spite of a present infection. This may occur due to an insufficient bacterium load (Hong et al., 2023), the involvement of a highly variable pathogen species with delayed growth cycles, or the influence of prior antibiotic therapy (Brückner et al., 2019).

In recent years, next-generation sequencing (NGS) has been reported as being used in the histopathological analysis of arthrofibrosis (Bayram et al., 2020). In addition, several NGS-based approaches have proven to have a high yield in the microbiological diagnosis of PJI (Henríquez et al., 2025; Hong et al., 2023). Nevertheless, the use of NGS specifically as a microbiological tool for detecting pathogens in arthrofibrosis cases has not been reported previously. In earlier studies that failed to link low-grade PJI and arthrofibrosis, Sanger sequencing was used (Brückner et al., 2019). More recent NGS techniques have demonstrated better performance than Sanger sequencing, particularly due to their ability to detect multiple microorganisms in polymicrobial infections (d'Humières et al., 2025; Olearo et al., 2025). Therefore, these negative findings may have been influenced by the sequencing technique employed (Sanger) and its lower sensitivity in the detection of microorganisms compared to advanced NGS platforms.

In conclusion, our study suggests a potential relationship between arthrofibrosis and PJI. These findings contribute to the growing body of evidence that suggests that low-grade infections may contribute to the development of arthrofibrosis. If these results are confirmed by further research, this would change the management of these patients, requiring a similar approach to PJI. Even if the relationship is not causal, considering low-grade PJI as a contributing factor and actively ruling it out in cases of arthrofibrosis may improve clinical outcomes and patient prognosis.

Our study has several limitations. First, it is a retrospective study with a relatively small sample size, as only 14 samples were collected from 17 patients diagnosed with arthrofibrosis. The relatively small sample size and the possibility of selection bias are important considerations that may affect the generalizability of the findings. Additionally, the absence of a control group is a limitation that restricts the ability to account for confounding factors and should be considered in future studies. Certain factors potentially related to infection but not included in the EBJIS criteria were not evaluated in this study. These factors include prior administration of pre-operative or intraoperative antibiotics, the presence of biofilm-associated “persister” organisms, and the inherent limitations of culture and even molecular diagnostics (e.g. PCR), which may contribute to false-negative results and complicate data interpretation. Moreover, detection by molecular biology methods, i.e. PCR or NGS, was not performed. Prospective studies with lager cohorts employing molecular biology techniques capable of detecting non-viable, difficult-to-isolate, or low-load microorganisms are necessary to confirm the hypothesized association between arthrofibrosis and low-grade infections. Upcoming NGS data may fully decipher the link between low-grade PJI and arthrofibrosis. Finally, the follow-up period of 1 year is relatively short and may be insufficient to fully exclude the presence of low-grade PJI.

## 5 Take-home messages

1. Low-grade PJI may play a contributory role in the development of arthrofibrosis.
2. Further studies involving larger cohorts and incorporating molecular biology techniques are needed to elucidate the role of these microorganisms in arthrofibrosis. Microbiological, biochemical, anatomical pathology, and clinical data of patients grouped according to their microbiological outcomes are shown in Table 3.
3. The identification of low-virulence microorganisms in cases of arthrofibrosis could have significant implications for its clinical management and treatment strategies.

## Data Availability

The data used to conduct this study are patient data from our institution. All data were collected and stored securely within database software that is used by our institution; for this reason, no additional data are available.
